# Hydrogen Peroxide: A Ubiquitous Component of Beverages and Food

**DOI:** 10.3390/ijms26073397

**Published:** 2025-04-05

**Authors:** Izabela Sadowska-Bartosz, Grzegorz Bartosz

**Affiliations:** Laboratory of Analytical Biochemistry, Institute of Food Technology and Nutrition, Faculty of Technology and Life Sciences, University of Rzeszow, 4 Zelwerowicza Street, 35-601 Rzeszow, Poland; gbartosz@ur.edu.pl

**Keywords:** hydrogen peroxide, tea, coffee, honey, milk, medicinal herbs, spices, alcoholic beverages, antimicrobial activity

## Abstract

Hydrogen peroxide (H_2_O_2_) plays a signaling role in the body. Numerous studies demonstrated that H_2_O_2_, generated mainly by autoxidation of polyphenols, ascorbate and other reduced compounds, is a common component of beverages such as honey, tea, coffee, formulated drinks and alcoholic beverages, and is generated in cooked vegetables. It is produced in fresh milk predominantly by xanthine oxidase. The antimicrobial action of honey depends mainly on H_2_O_2_ generated by glucose oxidase and polyphenol autoxidation. Many components of beverages and food scavenge generated H_2_O_2,_ so its level is a result of the balance between generation and scavenging. This review discusses the mechanisms of hydrogen peroxide formation, collects evidence for the presence and generation of H_2_O_2_ in beverages and food, discusses its fate in the gastrointestinal tract, evolutionary aspects of human exposure to alimentary hydrogen peroxide, and both adverse action and possible beneficial effects of the consumed hydrogen peroxide.

## 1. Introduction

Hydrogen peroxide (H_2_O_2_), a product of two-electron (per molecule) oxygen reduction, is an oxidizing agent with a standard redox potential at pH 7 E◦’(H_2_O_2_, H^+^/H_2_O, HO^−^) of 0.320 V and a weak reducing agent, with an E◦’ (O_2_^•−^, 2H^+^/H_2_O_2_) of 0.940 V. Hydrogen peroxide is used, i.a., as a wound antiseptic [1,2] and as a bleaching agent in dentistry [3,4]. These applications require high concentrations of hydrogen peroxide of an order of 1 M or higher. At the other concentration extreme, hydrogen peroxide, produced by aerobic cells to nano- to low-micromolar concentrations, is a key secondary messenger signaling molecule in the regulation of many biochemical reactions [5,6,7].

The signaling role of H_2_O_2_ is based on its reactions with proteins harboring redox-sensitive elements, such as reactive cysteine residues and metal centers. Oxidation of these structures controls activities of proteins such as protein phosphatases, kinases or transcription factors referred to as redox switches, key players in the regulation of biochemical pathways. A change in the concentration of hydrogen peroxide results in a change in the oxidation state of a redox switch, regulating a downstream pathway and transducing the information along a signaling cascade [6,8,9].

Hydrogen peroxide is removed in vivo mainly in enzymatic reactions catalyzed by catalases, peroxidases (in animals chiefly glutathione peroxidases), peroxiredoxins and in non-enzymatic reactions with available reducing compounds [10,11,12].

Somewhat surprisingly, hydrogen peroxide is also a ubiquitous component of environmental and laboratory water, is commonly generated in beverages and food and is consumed every day.

## 2. Hydrogen Peroxide in Environmental and Laboratory Water

Hydrogen peroxide is a natural component of any water reservoir; its contents in wet precipitates (rain and ice core samples) water were reported within the range of 10^−8^–10^−5^ M. Different authors reported values of H_2_O_2_ concentrations in atmospheric precipitation of up to ca. 200 μM, but usually below 50 μM [13]. In rainwater collected at the roof level in central Kowloon, China, the H_2_O_2_ concentration was 15.9 μM [14]. Similar concentrations (0.13–48.4 μM) were found for rainwater in Wilmington, NC, USA [13] and in Miami, FL, USA (0.3–38.6 μM) [15]. Somewhat lower values (1.3–3.2 μM) were reported from Xi’an, China [16]. Rainwater in a typical mid-size city in tropical Brazil contained 5.8–96 μM H_2_O_2_ [17]. The sources of hydrogen peroxide in atmospheric waters include the dissolution of gas-phase hydrogen peroxide and aqueous-phase production [13]. A strong seasonal dependence of the rainwater H_2_O_2_ levels was observed, with the highest concentrations in the summer and lowest in the winter, correlating with the stronger solar radiation and higher vaporization of volatile organic compounds in the summer and fall. Measurements also showed a trend for an increase in the H_2_O_2_ levels with increasing ambient rainwater temperature [13,15]. Environmental pollution, including biomass burning emissions and photochemical reactions, were also found to be important factors affecting the concentration of H_2_O_2_ in the atmosphere and rainwater [17].

The reported concentrations of hydrogen peroxide in fresh and saline natural surface waters were lower, generally 0.01–5 μM [18], and lower in seawater in comparison to freshwater [19]. Seawater in the South China Sea was found to contain 14–24 nM H_2_O_2_ [20]. After rainfall, the H_2_O_2_ contents of the ocean surface sharply increased (10–100 times) but returned to a stationary level within several hours [21]. Shallow lakes typical of eastern North Carolina contain about 0.20 μM H_2_O_2_ on average, but extreme rains might triple the amount normally present [13]. Water from the unpolluted Volga river was found to contain about 1 μM hydrogen peroxide, with daily concentrations being determined by solar illumination, affecting both photochemical and biological generation, increasing in the daytime and decreasing at night [21].

Hydrogen peroxide can also be detected in laboratory water. Physical factors such as heat or mechanical shaking may induce the formation of minute amounts of H_2_O_2_ in aqueous solutions. Air-saturated 1 mM phosphate buffer, pH 6.8, was found to contain 5.7 nM H_2_O_2_ at room temperature. Heating the buffer at 75 °C for 4 h increased this level to ca. 8.5 nM [22]. Mechanical oscillations (30 Hz, amplitude of 5 mm) were reported to induce H_2_O_2_ formation in air-saturated water at a rate of 1 nM/min [23,24]. Hydrogen peroxide (ca. 30 μM) was found to be spontaneously produced from pure water by atomizing bulk water into microdroplets (1 to 20 μm in diameter), most probably via the recombination of hydroxyl radicals (^•^OH) generated by the loss of an electron from OH^−^ at or near the surface of the water microdroplets [25].

## 3. Sources of Hydrogen Peroxide in Beverages and Food

Several sources of hydrogen peroxide in beverages and food should be considered. One is the enzymatic production of hydrogen peroxide in fresh, not processed, food. In fresh milk, the main source of hydrogen peroxide is xanthine oxidase [26], while in honey, H_2_O_2_ is generated by glucose oxidase [27]. Generally, the main source of hydrogen peroxide in beverages and food is the autoxidation of food components. These compounds, such as phenolic compounds, ascorbic acid, thiols and reduced metal ions act as antioxidants, but when in contact with oxygen they are subject to oxidation (often referred to as autoxidation), eventually producing hydrogen peroxide [28].

The autoxidation of phenolic compounds, including those present in food and beverages, proceeds in two steps. In the first step, a phenolic compound H_2_Q is oxidized to a semiquinone free radical HQ^•^ in a reaction coupled to the reduction of molecular oxygen to the superoxide anion radical O_2_^•−^ (1); then, the semiquinone is oxidized to quinone Q, producing the second superoxide radical (2):

H_2_Q + O_2_ → HQ^•^ + O_2_^•−^ + H^+^ (1)

HQ^•^ + O_2_ → Q + O_2_^•−^ + H^+^ (2)

Dismutation of superoxide radicals (3) or the oxidation of another molecule by the superoxide radical (4) produces hydrogen peroxide:

O_2_^•−^ + O_2_^•−^ + 2H^+^ → H_2_O_2_ + O_2_ (3)

H_2_S + O_2_^•−^ + H^+^ → HS^•^ + H_2_O_2_ (4)

where H_2_S is an oxidizable substrate and HS^•^ represents a radical product of its one-electron oxidation [29].

The autoxidation of phenolics is pH-dependent, as the phenolate anion is more reactive than the undissociated phenol group. The production of H_2_O_2_ by epigallocatechin gallate (EGCG), the main catechin present in the tea increased drastically with the pH elevation from 6 to 8 being practically constant in the pH range of 2–6 and about 100 times lower than that, also practically constant, in the pH range of 8–12. Autoxidation of one EGCG molecule produces two molecules of hydrogen peroxide, although at higher EGCG concentrations, the amount of H_2_O_2_ formed is lower [30]. The autoxidation can be catalyzed by transition metal ions [31]. Removal of the transition metal by metal chelators (phenantroline, desferoxamine) decreased H_2_O_2_ formation by tea catechins [32].

Similarly, the autoxidation of other food components produces hydrogen peroxide, usually in two one-electron steps involving superoxide radical anion as an intermediate. The relatively low standard reduction potential of ascorbate (0.19 V for the dehydroascorbate/ascorbate couple at pH 3.5) should allow it to be readily oxidized by molecular oxygen. However, although this redox reaction is thermodynamically possible, it is spin-forbidden and the only ascorbate species capable of true autoxidation is the ascorbate dianion whose share rises with increasing pH, and thus ascorbate autoxidation rate increases with pH elevation. Ascorbate autoxidation is also catalyzed by transition metal ions [33,34,35].

Low-molecular weight thiols and cysteine residues in proteins, as well as reduced free and protein-bound metal ions and other components of beverages and food, are also subject to autoxidation when in contact with oxygen. As the thiolate anion is more reactive than the not dissociated thiol, the rate of thiol autoxidation of thiols is again pH-dependent and enhanced by alkaline pH and may be catalyzed by metal ions [28]. Organic acids such as tartaric acid in wine can be subject to iron-catalyzed autoxidation [36,37]. Autoxidation of Maillard reaction products formed in reactions of sugars and amino acids at elevated temperatures was suggested to be a significant source of H_2_O_2_ in the coffee [38].

Hydrogen peroxide formed in the autoxidation reactions can oxidize further substrates [28,37], thus perpetuating the oxidation of beverage/food constituents.

## 4. Hydrogen Peroxide in Beverages and Food

### 4.1. Milk

Mother’s milk is not only healthy, and initially the only nutrient for a newborn, but it also has antibacterial, antiviral, antiamebal and antifungal properties. One of the main antimicrobial components of the milk is hydrogen peroxide, which has a microbicidal action itself and is a substrate for lactoperoxidase. In human milk, the highest H_2_O_2_ concentration was found in the first week postpartum (25.0 µM), decreasing progressively during the second, third and fourth week postpartum to 20.4, 15.8 and 8.8 µM, respectively. Storage at freezing temperature for up to 4 weeks did not cause any significant decrease of the H_2_O_2_ concentration in the milk [39]. Another study found H_2_O_2_ concentration in fresh breast milk samples to be 27.3 µM [40].

Xanthine oxidase (XO; EC 1.17.3.2) is the main source of hydrogen peroxide in breastmilk. Xanthine oxidase activity was detected in the milk of all mammals studied, with higher activity in bovine milk than in human milk. XO is a major protein component of the milk fat globule membrane surrounding the fat droplets that form a suspension in freshly expressed milk. XO activity in breast milk samples was found to be 8.0 U/L. No XO activity was detected in pasteurized breastmilk, in infant dried-milk formula or in pasteurized bovine milk, demonstrating that milk XO is inactivated by commercial heat-pasteurization. In the presence of its biochemical substrates (hypoxanthine or xanthine), XO produces superoxide and hydrogen peroxide [41]:

Hypoxanthine + O_2_ + H_2_O → xanthine + H_2_O_2_ (5)

Xanthine + 2 O_2_ + H_2_O → uric acid + 2 O_2_^−•^ + 2 H^+^ (6)

Xanthine + O_2_ + H_2_O → uric acid + H_2_O_2_ (7)

Superoxide radical anion dismutates (reaction 3), producing hydrogen peroxide.

Milk XO can produce additional hydrogen peroxide in the mouth as saliva provides its substrates. When breastmilk was mixed with a neonatal saliva (containing endogenous 30 μM xanthine and 70 μM hypoxanthine), the interaction yielded an additional 40 μM H_2_O_2_ during a 1-h incubation [40].

Hydrogen peroxide may be added to the milk as an antibacterial preservative, although the supplementation of milk with H_2_O_2_ is prohibited in many countries due to its toxic effects. When added to milk and other foodstuff, H_2_O_2_ causes a series of negative effects and adversely affects nutritional value due to the oxidation of vitamins E and A and folic acid. Consequently, if ingested, H_2_O_2_ can cause adverse effects on the population’s health, especially for immunocompromised people. Concentrations of peroxide ions (O_2_^2−^) higher than 0.1% *w*/*w* may be the cause of cancer in mice and produce genotoxicity [42]. However, in countries where appropriate cooling of milk during preservation and transport may be difficult, prolongation of milk shelf-life by addition of hydrogen peroxide seems to be a method of choice. Addition of H_2_O_2_ up to the concentration of 0.14% was recommended to preserve the consumption fitness of raw cow’s milk [43].

Guidelines for admissible hydrogen peroxide content in milk levels of usage vary between countries. For example, in Australia and New Zealand, H_2_O_2_ up to 5 ppm (147 µM) is allowed for processing selected dairy-based products [44]. In Taiwan, H_2_O_2_ is only allowed in the processing of selected products, including surimi [45], while in Japan, no residual H_2_O_2_ is allowed in any ready-to-eat food product [44] (although its spontaneous formation is unavoidable as shown later on). In the USA, H_2_O_2_ is used in aseptic packaging provided the final concentration in the food is <0.5 ppm (14.7 µM) [44]. According to the Food and Drug Administration, the percentage of hydrogen peroxide in the milk used for the production of cheeses may not exceed 0.05% of the weight of the milk in the United States (only for the production of cheeses, but H_2_O_2_ must be eliminated during the pre-treatment step) [42,46].

### 4.2. Honey

A broad range of H_2_O_2_ concentrations was reported in honeys of various origin (0.04 to 4 mM, with an average of 2.5 mM [27]). Hydrogen peroxide in honey is produced mainly by glucose oxidase (EC 1.1.3.4) secreted into the honey by bees [47,48,49]. The levels of hydrogen peroxide in honey are determined by the difference between the rate of its production and its destruction, mainly by catalases but also by reactions with polyphenols [49]. Bees introduce glucose oxidase to honey during nectar harvesting; the *gox* gene coding for this enzyme is solely expressed in the hypopharyngeal glands of worker bees. The amount of glucose oxidase in the honey depends on the age and health status of the foraging bees [48]. Catalase originates from pollen and its level depends on the amount of pollen grains in the honey [47]. The colloidal structure of honey was postulated to facilitate H_2_O_2_ formation by increasing the activity of glucose oxidase [47,50]. However, some studies suggest a lack of correlation between levels of H_2_O_2_ and glucose oxidase activity in honey [51]. Manuka honey, a pronounced medical-grade honey, accumulates only negligible amounts of H_2_O_2_. This may be explained by the activity of methylglyoxal, a major antibacterial factor of manuka honey, which induces a modification of protein components of the honey, including glucose oxidase, resulting in a loss of their biological activities. The addition of methylglyoxal dose-dependently inhibited H_2_O_2_ production in honeydew honey [52].

Enzymes contained in flower nectar may also produce hydrogen peroxide. Microorganisms, such as fungi and bacteria, found in some honeys, can contribute to H_2_O_2_ generation as well. Polyphenol (flavonoid and phenolic acid) autoxidation may be another source of hydrogen peroxide in honey [47]. Darker honeys usually have higher total polyphenol content [27,53]. The production and accumulation of hydrogen peroxide was the highest in dark honeys, intermediate in medium dark honeys and the lowest in light honeys, which suggests that compounds influencing honey color participate in H_2_O_2_ production [27]. Although several groups of compounds, among them carotenoids, polyphenols, metal ions and melanoids, can contribute to the color of honey, the ability of phenolic compounds to autoxidize makes them the most probable non-enzymatic generators of H_2_O_2_. Flavonoids and their quinones are themselves natural chromophores displaying a range of colors from light yellow to dark brown; the colors are influenced by interaction with iron ions [54]. Moreover, the flavonoid oxidation to semiquinones and quinones generates yellowish colors, while oxidatively polymerized phenolic acids and flavonoids increase browning [27].

Dark honeys were found to possess significant concentrations of *p*-hydroxybenzoic, chlorogenic and caffeic acids in addition to phenyllactic, *p*-coumaric, abscisic and ellagic acids [55,56,57]. They also contain flavonoids, including pinocembrin, galangin and myricetin derivatives, glycosylated flavonols, quercetin rhamnoside, rutin and kaempferol rhamnoside, chrysin (5,7-dihydroxyflavone), quercetin, kaempferol, fisetin, myricetin, morin, and rutin, daidzein and genistein [55,57,58]. Cooperative action between flavonols (quercetin or rutin) and flavanones (naringenin and naringin) which increases the ability of flavanones to donate electrons and oxidize has been demonstrated [59]. Thus, the synergistic action of polyphenolic acids and flavonoids rather than an individual compound was suggested to play a role in the pro-oxidant activity and generation of H_2_O_2_ in honey. The average pH of honey ranges between 3.5 and 5.5, which should be inhibitory for polyphenol autoxidation. However, several aspects of polyphenol autooxidation can compensate for the effect of low pH [27,50].

Similarly, additional mechanisms contribute to the degradation of hydrogen peroxide in honey, including ascorbic acid, which may be oxidized by hydrogen peroxide. Hydrogen peroxide can also be consumed in the Fenton reaction [27]. The presence of trace metals bound to proteins or chelated by polyphenols has been demonstrated in honeys. Dark honeys (buckwheat, heather, and honeydew in comparison to light honeys such as acacia or rape honey) were found to contain higher quantities of transition metal ions such as Fe, Zn, Cu, and Mn [60,61].

The dilution of honey activates glucose oxidase (and also phenolic autoxidation) resulting in time- and dilution-dependent production of H_2_O_2_. Honey diluted to 20–40% was reported to generate hydrogen peroxide, which reached a maximum concentration of 5.62 mM for 30% honey after 24 h [62]. Another study, using four West Australian honeys, revealed maximum H_2_O_2_ generation in 30% honey solution in the range of 76–479 µM after 6 h. No H_2_O_2_ was generated in the Manuka honey [63]. Honeydew honey samples diluted to 40% produced 0.3 to 3.4 mM H_2_O_2_ after 24 h incubation at 37 °C [64].

Generally, the relationship between H_2_O_2_ production and honey dilution assumes an inverted U-shaped curve [50,51]. This effect is of physiological significance, as honey is consumed often as an additive to beverages; of course, its addition to hot tea inactivates glucose oxidase, lowering this effect; additionally, honey is diluted in the digestive tract which may activate glucose oxidase (if active) until inactivated and digested. A similar phenomenon can occur in honey dressings used for wound healing [27]. What is important for the assay of H_2_O_2_ levels in honey is that such an effect may also occur during the assay of hydrogen peroxide content in the honey, since honey samples are usually diluted prior to the assay, and may lead to some overestimation of the real H_2_O_2_ concentration in the honey, dependent on details of the assay procedure.

Standard heat and filtration processing (heating at 45 °C for 8 h and filtering through a 100 µm filter (Millipore, Burlington, MA, USA) were found to reduce the levels of H_2_O_2_ in honey [65].

### 4.3. Tea and Coffee

Fresh tea leaves contain 18–32% dry weight of flavan-3-ols. Epigallocatechin gallate constitutes about half of this amount, followed by epigallocatechin, epicatechin gallate, epicatechin, gallocatechin, catechin and other minor flavanols and other polyphenols, mainly gallic acid [66,67]. Coffee is also rich in polyphenols (up to 1–2% of dry weight), including phenolic acids and flavonoids, first of all EGCG [68,69]. Black tea also has significant amounts of theaflavins, thearubigins and other catechin polymeric pigments [70].

Coffee contains significant amounts of Maillard reaction products [38,71]; some amounts of these compounds are also present in processed tea [72]. All these compounds can generate hydrogen peroxide upon autoxidation. The presence of the 1,2,3-trihydroxybenzene structure (a galloyl group) or the *o*-3′, 4′-dihydroxy catechol structure determines the maximal electron-donating effect of polyphenols and autoxidation to corresponding quinones [73]. The presence of transition metal catalyzing the autoxidation of polyphenols is essential [74]. Differences in the concentrations of Fe (II), Cu (I) and Mn (II) transition metals are thus important factors determining the yield of H_2_O_2_ formation in tea and other beverages [75].

Solutions of catechins in pure water (100 µM) at 50 °C produced H_2_O_2_ with the following sequence of yields during 2-h incubation: EGCG (127.0 µM) > epigallocatechin (51.5 µM) > epicatechin gallate (8.4 µM) > epicatechin (6.2 µM) > gallic acid (1.4 µM) [76]. In another study, the sequence of H_2_O_2_ yields by 500 µM catechins (37 ˚C, 1 h) was generally similar: EGCG (191 µM) > epigallocatechin (179 µM) > gallic acid (28 µM) > epicatechin (21 µM) > methyl gallate (4 µM) [77].

1,2,4-Benzenetriol (hydroxyhydroquinone) is one of the main components in roasted coffee which generates H_2_O_2_. Although it is stable in the slightly acidic brewed coffee, it generates 0.5–0.6 moles of hydrogen peroxide per mole during incubation under aerobic conditions at pH 7.4 [78].

Although the average pH of tea and coffee is around 5.5 and does not promote the deprotonation of polyphenol -OH groups, tea and coffee, the most commonly consumed beverages next to water, can generate hydrogen peroxide to achieve levels over 100 μM, in contrast to cocoa, which was found not to generate H_2_O_2_ [79]. However, hydrogen peroxide was also reported in cocoa (infused with water and not containing milk) [80].

Hydrogen peroxide generation was much higher in a phosphate buffer, pH 7.4, than in distilled water (green tea, 1102 µM vs. 0 µM pH 6.0, black tea, 790 µM vs. ca. 210 µM at pH 5.5; coffee, 240 µM vs. ca. 140 µM at pH 6.8) [81]. While green tea (1% *w*/*v*) in distilled water was found to generate only 19 μM H_2_O_2_ upon 2-h incubation, this tea added to a cell culture medium (Dulbecco’s Modified Eagle’s Medium, DMEM) of neutral pH and containing some Fe (III) generated up to 180 μM H_2_O_2_ [82]. Green tea and several herbal teas generated considerable amounts of H_2_O_2_ when brewed in a phosphate buffer, pH 7.4 (up to ca. 1.1 mM for green tea). The addition of hibiscus and thorn apple tea decreased the production of H_2_O_2_ in the catechin-rich green tea, possibly due to a lowering of the pH of the mixture [83,84,85]. In another study, green tea produced up to 100 µM of H_2_O_2_ during 3-h incubation. More H_2_O_2_ was generated in tea prepared with tap water than in tea prepared with distilled water. The addition of a lemon slice decreased H_2_O_2_ generation in the tea, partly due to the lowering pH of the tea [32]. Hydrogen peroxide was produced in various tea brands and tea-like drinks, including white, green, red and black tea, rooibos and yerba mate [80].

Espresso coffee, filter coffee and instant coffee were found to generate hydrogen peroxide upon brewing (ca. 80, 20 and 15 µM, respectively). The H_2_O_2_ concentrations remained fairly stable at room temperature for up to 5 h; however, 24-h incubation under cell culture conditions (37 °C, 5% CO_2_) led to a significant increase in the H_2_O_2_ concentration, up to ca 370 and 330 µM for filter coffee and espresso, respectively. Green coffee did not generate measurable amounts of H_2_O_2_ upon brewing and subsequent incubation at room temperature but produced low amounts of hydrogen peroxide (ca. 20 µM) when incubated under cell culture conditions [38].

Only small amounts of hydrogen peroxide were detected in commercially sold bottle-packaged beverages, such as tea or coffee, immediately after opening of bottles, but H_2_O_2_ was gradually produced in the beverages after opening the caps, i.e., upon exposure to air. The production of H_2_O_2_ increased both with the duration of the exposure to air and the rise in temperature. The addition of citric acid, malic acid, succinic acid, fumaric acid, L-ascorbic acid, L-glutamic acid and L-aspartic acid to the tea reduced H_2_O_2_ generation, apparently due to the lowering of the pH. The addition of milk or lemon to the black tea reduced H_2_O_2_ generation but milk did not affect H_2_O_2_ generation by coffee. Hydrogen peroxide production in the green tea was lowered by the addition of aqueous extracts of citrus (orange, grapefruit, natsumikan, lemon or lime) juices and peels. While the effect of citrus juices was mainly due to the lowering of pH, the peels decreased H_2_O_2_ production even in buffer, pH 7.4. These extracts were also able to degrade H_2_O_2_ preformed in the tea, which may partly explain their effect [84,85].

Linalool, one of the substances responsible for the aroma of the tea, increased the H_2_O_2_ production by EGCG, most likely due to a combined action of flavanols and other tea components. Hydrogen peroxide destroyed linalool, leading to flavor deterioration of tea beverages [76].

Holding green tea in the mouth or chewing tea leaves generates hydrogen peroxide in the saliva. Holding 0.1–0.6% green tea in the mouth for 2 min generated 2.9–9.6 µM H_2_O_2_ while chewing 2 g of green tea leaves produced up to 31.2 µM H_2_O_2_ [86].

Increased levels of H_2_O_2_ were also detected in the urine of healthy volunteers for up to 2 h after drinking coffee [87]. This phenomenon was confirmed in another study, which showed that drinking a cup of coffee resulted in the excretion of 0.5–5 µmol H_2_O_2_ per µmol creatinine during 3 h (1 to 4 h after consumption). Autoxidation of 1,2,4-benzenetriol (and other coffee polyphenols) was suggested as the main source of hydrogen peroxide. The hydrogen peroxide levels of urine increased 5-fold during storage for 2 h after urine collection, in line with this suggestion. Some hydrogen peroxide was detected in healthy human urine (100 ± 60 μM [88], 1–113 µM [89]) and it was noted that urinary hydrogen peroxide levels slowly increased when urine was allowed to stand in air, possibly due to autoxidation of polyphenols taken from food [89,90].

### 4.4. Cooked Vegetables

Vegetables contain compounds prone to autoxidation, especially phenolics and ascorbic acid. Hydrogen peroxide was found to be generated in cooked vegetables. The highest H_2_O_2_ concentration was found in the homogenate of broad bean (73–76 µM in 1:2, *w*/*v* homogenates in phosphate buffer, pH 7.4, 10-min cooking at 95 °C) followed by broccoli (19 μM), onion (10 μM) and leek (10.0 μM). In the decoctions, the highest concentration of hydrogen peroxide was revealed for broccoli (24 μM), broad bean (21 μM), carrot (13 μM) and cauliflower (13 μM). Cooking vegetables in tap water also generated H_2_O_2,_ albeit at lower concentrations (28 μM for broad bean). Incubation of the homogenates at room temperature decreased or increased the level of hydrogen peroxide after 3 h, depending on the vegetable, and decreased after 24-h incubation [91]. About 10 times more hydrogen peroxide was generated in homogenates of anthocyanin-rich black carrot in comparison with orange carrot [92].

### 4.5. Herbal Extracts and Spices

Extracts of *Rosa canina*, *Rhodiola rosea*, *Hypericum perforatum*, and *Gentiana lutea* were found to generate more H_2_O_2_ at pH 7.8 than at pH 6.0. The extract of *R. rosea* (50 µg/mL) produced about 8 µM H_2_O_2_ at pH 6.0 and over 100 µM H_2_O_2_ at pH 7.8 [93]. Generation of hydrogen peroxide was also demonstrated in infusions of *Achillea millefolium*, *Artemisia absinthium*, *Cistus incanus*, *Polygonum aviculare*, *Taraxacum officinale*, *Melissa officinalis*, *Mentha piperica*, *Plantago lanceolata*, *Tussilago farfara*, *Urtica dioica* and *Rosmarinus officinalis*, of leaves of *Betula pendula*, and inflorescence of *Calendula officinalis* and *Lavandula angustifolia* in tap water, without pH adjustment. The main source of hydrogen peroxide in herbal extracts, like in tea, is the autoxidation of phenolic compounds by a two-step mechanism (Section 3), as demonstrated by the presence of an electron spin resonance (ESR) signal of the semiquinone radical and generation of superoxide in tea and herbal infusions [80,94]. The extracts also scavenged exogenous H_2_O_2_ [93,94].

Generation of hydrogen peroxide was also detected in the infusions of spices (chili, allspice, green and black pepper, caraway seeds and coriander) [80].

### 4.6. Other Beverages

Hydrated protein drink mixes were reported to generate hydrogen peroxide, due mainly to ascorbate autoxidation [95]. Flavored carbonated soft drinks were found to contain up to 40–50 µM H_2_O_2_. Hydrogen peroxide was also detected in carbonated and noncarbonated mineral water at concentrations of up to several µM. Ascorbic acid was found to be the main component responsible for the generation of H_2_O_2_ in soft drinks; interaction with other compounds, such as lactate, glucuronolactone, dextrose, and citrate, may augment the production of hydrogen peroxide. Exposure to light was suggested to contribute to the in situ production of H_2_O_2_ in beverages during shelf storage [44]. Commercially available fresh and preserved orange juices were found to contain 2–11 µM H_2_O_2_. For the majority of juices, the concentration of H_2_O_2_ was significantly enhanced by aeration, particularly for the preserved juices, reaching a maximum level 1–5 h after opening, up to four times after aeration for 1 h; however, after 24 h the levels of H_2_O_2_ generally declined to baseline or below. The level of hydrogen peroxide correlated with the concentrations of ascorbic acid, total reducible substances and total sugar [96].

### 4.7. Unhealthy Providers of Pleasure: Alcoholic Beverages and Tobacco Smoke

Hydrogen peroxide is generated in wine mainly by the oxidation of phenolic compounds but consumed in reactions with SO_2_ and other wine components [97]. The autoxidation of polyphenols in wine, especially in the presence of sulfite, is significantly accelerated by transition metal ions [98]. Generation of hydrogen peroxide was demonstrated during accelerated wine oxidation by oxygen and associated with polymerized polyphenols [99].

No generation of hydrogen peroxide was found in two red wines but the addition of wine to DMEM resulted in the appearance of 71–84 µM H_2_O_2_ after a 2-h incubation [82]. A study of 40 wines demonstrated that H_2_O_2_ can be detected in some but not all wine samples, and that hydrogen peroxide can be both produced and scavenged by wines, i.a., by the sulfites present in the wine [100].

Many barley proteins are rich in thiol groups and although these exist in unmalted grain in the oxidized form, they become increasingly reduced during malting. These proteins autoxidize, producing hydrogen peroxide during mashing. Any hydrogen peroxide produced from malt constituents is rapidly consumed in reactions with polyphenols and enzymes such as catalase and peroxidases [101]. Hydrogen peroxide can also be produced by autoxidation of cysteine and cysteine residues in proteins catalyzed by Cu (II) during beer malting and brewing [44]. The presence of hydrogen peroxide was detected in six out of ten beers studied; the generation of hydrogen peroxide was found in 4-fold diluted beers incubated with air access [102].

Whiskies contain gallates and other phenolic compounds extracted from toasted, hard oak barrels. These compounds are prone to autoxidation. Fruit tinctures contain polyphenols and other autoxidizing compounds, such as ascorbic acid. The presence of hydrogen peroxide was found in whisky, brandy as well as cherry, raspberry, hazelnut, quince, apricot, plum, lemon, dogwood and curcuma tinctures. Hydrogen peroxide was also generated in most of these beverages upon bottle opening [102].

The presence of an ESR signal of free radicals, probably mainly of semiquinone radicals, products of the first step of polyphenol autoxidation, was detected in wines. The intensity of this signal increased after contact with air and then decreased [103]. An ESR signal of semiquinone radicals was detected in concentrated Scotch whisky [104]. The semiquinone radical as well as the generation of superoxide radical, was detected in beer, brandy and a fruit tincture [102].

Cigarettes are neither food nor beverages; nevertheless, smokers often combine sipping wine or beer with smoking, or smoking is for them a kind of dessert after a meal. It should be noted that cigarette smoke generates hydrogen peroxide in contact with an aqueous medium [105,106,107,108], probably due mainly to the autoxidation of polyphenols present in the smoke [106,107].

Concentrations of hydrogen peroxide found in environmental water, beverages and food and methods used for their estimation are listed in Table 1.

## 5. Scavenging of Hydrogen Peroxide by Beverages

As mentioned before, components of beverages not only produce hydrogen peroxide but also scavenge exogenous H_2_O_2_. Both generation and scavenging of hydrogen peroxide were demonstrated for pure polyphenols. While the concentration of hydrogen peroxide decreased in pyrogallol supplemented with H_2_O_2_, the generation of hydrogen peroxide prevailed over its scavenging in a gallic acid solution while in the case of quercetin the net effect depended on the H_2_O_2_ concentration, H_2_O_2_ scavenging prevailing at higher concentrations of exogenous hydrogen peroxide [80]. The yield of H_2_O_2_ generated by EGCG reached its maximum after 48 h of incubation; afterwards, with the extension of incubation time, the hydrogen peroxide produced in the system was gradually removed by EGCG or its degradation products, so that it eventually approached zero [76]. Green, white, red and black tea [84,124], herbal infusions [93,94] and wine [100] were demonstrated to scavenge exogenous H_2_O_2_. The ability of plant extracts to scavenge exogenous hydrogen peroxide was found to decrease with increasing pH (7.8 vs. 6.0) [93]. Milk was found to decrease the net H_2_O_2_ production by beverages and showed some ability to remove H_2_O_2_ itself [79], apparently enzymatically in the case of fresh milk (and not-too-hot beverages).

Thus, depending on the composition of a beverage, the hydrogen peroxide produced is partly or totally scavenged by the beverage, and the amount/generation rate of H_2_O_2_ measured depends on the net difference of the real rate of its generation and the rate of scavenging. Hydrogen peroxide may be generated even in beverages in which it is not detected; when ingested and diluted, these beverages may generate hydrogen peroxide as long as they are in contact with oxygen at near-neutral pH. The hydrogen peroxide generated under these conditions may react not only with the compounds present in the beverage but also with other substances present in the body.

## 6. Fate of Hydrogen Peroxide in the Digestive System

In the mouth, further production of H_2_O_2_ from beverages and food can proceed [86]. The ingested hydrogen peroxide may be metabolized by the salivary peroxidase and myeloperoxidase and decomposed by catalase [26,125,126]. Thiocyanate is considered to be an important peroxidase substrate. In neonate saliva the median concentration of thiocyanate was found to be 0.42 mM (with a wide range of 0.08–3.20 mM), then it declined over the weaning period before rising again to an adult median of 1.38 mM (0.45–5.82 mM) [40].

Residual hydrogen peroxide can be transported down the digestive tract. The epithelial cells of the stomach and intestines are always being reproduced, even when the superficial ones die, which may be a physiological adaptation to the exposure to noxious agents, including hydrogen peroxide.

At the low pH of the stomach, no production of hydrogen peroxide can be expected. The autoxidation of dietary components resulting in the production of H_2_O_2_ can be resumed in the neutral/alkaline environment of the intestine. At the colonic mucosa, oxygen partial pressure is below 25% of airborne oxygen content, which still allows for autoxidation, albeit with lower efficiency; then, the oxygen content falls further down the digestive tract [127].

## 7. Adverse Effects of Beverage and Food-Derived Hydrogen Peroxide

Consumption of hydrogen peroxide at concentrations > 3% is known to produce acute symptoms, including gastrointestinal irritation and bleeding burns in the duodenum and stomach, as well as renal failure and gas embolism of the lung and heart tissues [128,129]. Concentrations of hydrogen peroxide ingested in beverages are lower by 3–5 orders of magnitude but it has been hypothesized that the hydrogen peroxide derived from beverages can exert adverse effects including induction of gastric ulcers, gastrointestinal and duodenal cancers [44].

## 8. Possible Beneficial Effects of Dietary Hydrogen Peroxide

Dietary hydrogen peroxide can have a microbicidal effect, especially in terms of bactericidal action. Concentrated (0.8 to 8 M) hydrogen peroxide is commonly used to disinfect and sanitize medical equipment in hospitals. The bactericidal effectiveness of H_2_O_2_ has been demonstrated against various microorganisms including *Staphylococcus*, *Streptococcus*, *Pseudomonas* species, and *Bacillus* spores [46]. The antibacterial activity of such low H_2_O_2_ concentrations as those occurring in beverages has been questioned. Nevertheless, even low concentrations of hydrogen peroxide can give rise to a more reactive oxygen species, of undoubtable microbicidal action.

Xanthine oxidase in milk has been shown to catalyze the anaerobic reduction of inorganic nitrite to nitric oxide. Superoxide generated by xanthine oxidase in the presence of molecular oxygen reacts rapidly with nitric oxide to produce peroxynitrite, also a powerful antibacterial agent. Hydrogen peroxide is a substrate for lactoperoxidase present in both milk and saliva, producing antibacterial hypothiocyanite. Supplementation of the saliva–milk media with XO substrates, xanthine and hypoxanthine to activate the milk XO/LPO system significantly inhibited the growth of *Staphylococcus aureus* compared to the control and nucleoside-supplemented saliva. Inhibition of XO by oxypurinol restored normal growth [40].

The antibacterial, antimycotic and antiviral role of peroxidases has been demonstrated in in vitro studies and should also concern salivary peroxidases. Inhibition of the growth of oral *Streptococcus* by hydrogen peroxide in the presence of salivary peroxidase was far more effective than by H_2_O_2_ alone [130]. The presence of salivary peroxidase activity was associated with the presence of Gram-positive cocci microbial flora in the oral cavity, and its insufficiency has been linked to pathologies, such as caries, periodontitis or infections of the oral mucosa. Thus, oxidants generated by oral peroxidases appear to be an essential ecological determinant for oral health since they could have allowed for the selection of a symbiotic microbiota resistant to oxidative stress [131]. Hydrogen peroxide, the substrate for these peroxidases, is produced mainly by neutrophils and bacteria, but dietary H_2_O_2_ can contribute.

The healing properties of honey have been known for centuries but it was recently confirmed that honey can be effective for clearing infections in a wide range of wounds, including abscesses, surgical wounds, traumatic wounds, burns, and ulcers of varied etiology, especially those not responding to conventional therapy. While this effect seems to be mediated mainly by hydrogen peroxide [132], it is contributed to by other mechanisms. Hydrogen peroxide was identified to be the “inhibine”, a previously described component of honey that inhibited bacterial growth [133], although further studies brought complications to this simple picture. In any case, removal of H_2_O_2_ by pre-treatment of honey with catalase reduced the honey’s antibacterial activity by 50–80%, confirming its major contribution to its antibacterial activity [63,112,116,134]. The amount of H_2_O_2_ produced in honey significantly correlated with the honey Minimum Inhibitory Concentration (MIC) and Minimum Bactericidal Concentration (MBC) [47].

Hydrogen peroxide protects against *Helicobacter pylori* [135]. In mice, hydrogen peroxide produced physiologically by DUOX2 was demonstrated to prevent the infection and inflammation of the gastric mucosa by *Helicobacter felis* [136]. This action can be succored by dietary hydrogen peroxide. A retrospective cross-sectional study revealed that consumption of honey, coffee and green tea had protective effects against *H. pylori* infection [137]. All of them may contain or produce hydrogen peroxide.

Low concentrations of hydrogen peroxide transmitted to or produced in the intestines from beverages and food may play a role in the functioning of this part of the digestive tract. DUOX-derived H_2_O_2_ is required for control of intestinal microbiota in *Drosophila*. This oxidase generates a unique epithelial oxidative burst that limits microbial proliferation in the gut [138]. Hydrogen peroxide was demonstrated to play a signaling and regulatory role in the digestive tract [127]. NOX1-derived H_2_O_2_ induced by certain commensal bacteria, such as *Lactobacilli*, may contribute to mucosal wound healing through focal adhesions, enhancing epithelial cell migration and restitution after injury [139,140]. Low concentrations (1–5 μM) of lipid hydroperoxides were reported to promote the proliferation of colon-derived Caco-2 cells [141]. It can be expected that H_2_O_2_ may act similarly, as a similar pattern of cell growth stimulation was observed for low concentrations of various oxidants, including H_2_O_2_ [142]. Indeed, while high concentrations of hydrogen peroxide may damage colon cells, low H_2_O_2_ concentrations were found to stimulate colonic epithelial proliferation, thus contributing to epithelial repair [143]. Hydrogen peroxide was also reported to promote gastric motility [144]. Alimentary hydrogen peroxide may contribute to all these effects. It may also affect the composition of gut microbiome [145].

Moreover, in the intestines exogenous peroxide may react with available iron, forming hydroxyl radicals and other free radicals, which facilitate digestion as proteins subjected to free radical action may show enhanced susceptibility to proteolytic enzymes [146].

## 9. In Vitro Cellular Effects of Beverages Mediated by Hydrogen Peroxide

Many plant-derived beverages were found to exert cytostatic or cytotoxic effects on cells, especially malignant cells, in vitro. These effects were often partly mediated by hydrogen peroxide. The addition of green tea and red wine to the culture medium was dose-dependently cytotoxic to PC12 pheochromocytoma cells; this effect was considerably reduced by catalase [82]. Green tea and EGCG were found to increase the intracellular oxidation of dichlorodihydrofluorescein and DNA damage in RAW 264.7 macrophage-like cells and HL60 promyelotic leukemic cells; these effects were prevented by catalase but not superoxide dismutase [147]. Coffee reduced the viability of bovine aorta endothelial cells; catalase partly prevented this effect [38]. Pyrogallol, gallic acid and quercetin decreased the viability of DU-145 prostate cancer cells; this effect was alleviated by catalase [32]. Growth inhibition of PEO1 ovarian cancer cells by extracts of *Cistus*, *Gingko* and *Tilia* was partly prevented by catalase [94].

In vivo, autoxidation of polyphenols and other food components, if ingested, is vastly reduced due to the lower oxygen concentration while hydrogen peroxide produced is efficiently enzymatically scavenged. Therefore, many of the results of in vitro studies of the inhibition of growth of malignant cells by tea and other beverages overestimate the effect which can be achieved in vivo.

## 10. Evolutionary Aspects of the Generation of Hydrogen Peroxide in Beverages and Food

The presence of hydrogen peroxide in milk made people acquainted with the ingestion of hydrogen peroxide from the very beginning of evolutionary existence of our mammalian ancestors. Apparently, very early our protoplasts had contact with hydrogen peroxide generated in honey. However, more intensive exposure to hydrogen peroxide in food and beverages must have taken place after the mastering of fire and preparation of cooked food and beverages.

An increase in teeth size (megadonthy) before 3 million years ago (Ma) is attributed to a change in food consumed by hominoids, and inclusion of new food such as grasses and sedges [148,149]. From about 2.6 Ma, increased meat eating is well evidenced by archaeological sites that link stone tools and cut-marked bones [150]. The new foods were hard to digest, so the mastering of fire and cooking about 1.7 Ma by *Homo erectus* greatly increased their digestibility. This could have come with *Homo erectus* at about 1.7 Ma [149,151].

No significant amounts of H_2_O_2_ are ingested in fresh food due mainly to enzymatic decomposition, except for limited autoxidation of its components in the intestines, after deactivation of enzymes present in the food. In contrast, consumption of cooked food and beverages involves the ingestion of hydrogen peroxide produced by autoxidation of its components, especially at elevated temperatures and not removed by heat-denatured enzymes. Thus, there are reasons to postulate that our ancestors were dealing with hydrogen originally present in cooked food and beverages for at least 1.7 Ma. This period seems long enough to allow for the evolutionary acquisition of means of defense if alimentary hydrogen peroxide was really harmful. Comparison of the expression of H_2_O_2_-decomposing enzymes in the digestive tract of humans and their closest evolutionary relatives could shed light on this intriguing question.

## 11. Conclusions

Hydrogen peroxide is a ubiquitous component of beverages and plant-derived food, considered as the healthiest diet components, starting from breastmilk and honey, through tea and infusions of medicinal herbs and spices, till cooked vegetables. Studies on the generation of hydrogen peroxide in animal-based food are needed. Hydrogen peroxide may be harmful at high concentrations but the low concentrations of H_2_O_2_ ingested in the diet can exert positive health effects, including antimicrobial action and a signaling role in the digestive tract.

## Figures and Tables

**Table 1:** Reported concentrations of hydrogen peroxide in environmental water, beverages and food.

Material	Method	H_2_O_2_ Concentration	Reference
Rainwater in Miami, FL, USA	Spectrophotometric with HRP and N,N-diethyl-*p*-phenylenediamine (OPDV)	6.9 μM (0.3–38.6 μM)	[15]
Rainwater collected at roof-top level in central Kowloon, China	Spectrophotometric with HRP and OPDV	15.9 μM	[14]
Rainwater collected in Wilmington, NC, USA	Fluorimetric, with HRP and scopoletin	12 μM (0.13–48.4) μM	[13]
Rainwater (Xi’an, China)	Fluorimetric, with thiamine and Hb	1.3–3.2 μM	[16]
Ribeirão Preto, Brazil	Fluorimetric, with 2′,7′-dichlorofluorescin	28.6 ± 1.4 μM (5.8–96 μM)	[17]
Seawater (South China Sea)	Fluorimetric, with scopoletin	14–24 nM	[20]
Fresh and saline natural surface waters	Iodometric titration	0.05–5.00 μM	[18]
Human milk, 1st week postpartum	Luminol chemiluminescence	25.0 µM	[39]
Human milk, 1 to 5 days postpartum	Fluorimetric, with Ampliflu Red and HRP	27.3 μM	[40]
Bovine milk from a supermarket (Wuhan)	Fluorimetric with Fe^2+^ and coumarin	0.39–0.44 μM	[109]
Sterilized bovine milk	Electrochemical sensor using Hb–DNA/ZrO_2_/Au electrode	Not detected	[110]
Milk from a local market (Brazil)	Fe^2+^/thiocyanate/smartphone camera	Not detected	[111]
Honey, 18 kinds (Canada)	Fluorimetric, Ampliflu Red kit with HRP	29-239 µM	[112]
Various honeys, Australia	Spectrophotometric, with *o*-dianisidine and HRP	0–916 µM	[65]
Honey, 20 kinds (Slovakia)	Fluorimetric, Ampliflu Red kit with HRP	0.3–3.7 mM	[48]
Honeydew honey, mainly from *Abies alba* (Slovakia), 2 y old	Fluorimetric, Ampliflu Red kit with HRP	307 and 496 µM	[52]
Robinia (*Robinia pseudoacacia*) honey (Slovakia), 1 y old	Fluorimetric, Ampliflu Red kit with HRP	36.3 µM	[52]
Manuka honey, mainly from New Zealand, 3 y old	Fluorimetric, Ampliflu Red kit with HRP	78.9 µM	[52]
Manuka honeys from Australia and New Zealand	Spectrophotometric, with *o*-dianisidine and HRP	0–78 µM	[113]
Honey from *Leptospermum* spp. (Australia)	Spectrophotometric, with *o*-dianisidine and HRP	143 µM	[113]
Acacia, linden, rapeseed and sunflower honey	Megazyme GOX assay (absorptiometric)	12.1, 19.9, 12.3 and 37.2 µg/g, respectively	[114]
*Melilotus albus* honey, Poland	Spectrophotometric, with *o*-dianisidine and HRP	19–125 µM	[115]
Buckwheat honey, Poland	Spectrophotometric, with *o*-dianisidine and HRP	0.04–0.74 mM	[116]
Various honeys, Slovakia	Megazyme GOX assay (absorptiometric)	0.36–1.09 mmol/kg	[114]
Various honeys, Crete	Spectrophotometric, with *o*-dianisidine and HRP	36–45 µM in 30% *v*/*v* aqueous honey solution	[117]
*Acacia* spp. and *Ziziphus spina christi* honeys, Saudi Arabia	Ce(IV) sulfate titration	2.5 and 3.9% (ca. 0.73 and 1.15 M), respectively	[118]
35 monofloral honeys	Modified FOX assay (absorptiometric)	5.9–214.2 µmol/kg	[119]
24 honeys, Spain	Fluorescence Assay Kit using Ampliflu Red	21–1398 µmol/kg	[120]
Green tea, 1% (*w*/*v*) in distilled water	FOX assay (absorptiometric)	13 and 19 µM after 1 and 2 h, respectively	[82]
Green tea, 5 mg/mL phosphate buffer, pH 7.4, 24 h, 37 °C	FOX assay (absorptiometric)	1102 µM	[81]
Black tea, 5 mg/mL phosphate buffer, pH 7.4, 24 h, 37 °C	FOX assay (absorptiometric)	790 µM	[81]
Green tea and, rose, rosemary, sage, thyme and peppermint herbal teas in phosphate buffer, pH 7.4, 24 h incubation	FOX assay (absorptiometric)	ca. 1.1, 0.4, 0.3, 0.4, 0.25 and 0 mM, respectively	[84]
Oolong tea, black tea, coffee, frozen dried coffee in PET bottles, freshly opened	FOX assay (absorptiometric)	0.03–0.07 mM, ca. 0.05 mM, ca. 0.04 mM and ca. 0.1 mM, respectively	[83]
Oolong tea, black tea, coffee, frozen dried coffee in PET bottles, after 24-h incubation at 25 °C	FOX assay (absorptiometric)	0.22–0.44 mM, ca 0.54 mM, ca. 0.36 mM and ca. 0.35 mM	[83]
Green tea packed in a PET bottle (Japan), 24 h after opening	FOX assay (absorptiometric)	665 ± 22 µM	[85]
Green tea, 1 bag/200 mL of distilled or tap water, 3-h incubation, 21 °C	FOX assay (absorptiometric)	25 and 88 µM, respectively	[32]
Green, white, black and red tea, 15 min after brewing	FOX assay (absorptiometric)	53, 50, 73 and 47 µM, respectively	[80]
Rooibos, yerba mate and cocoa, 15 min after brewing	FOX assay (absorptiometric)	54, 67 and 38 µM, respectively	[80]
Coffee; thyme and rosemary extracts, 15 min after brewing	FOX assay (absorptiometric)	44, 53 and 42 µM, respectively	[80]
Coffee, 5 mg/mL phosphate buffer, pH 7.4, 24 h, 37 °C	FOX assay (absorptiometric)	240 µM	[81]
Twelve preparations of coffee, freshly prepared	Spectrophotometric, with Phenol Red and HRP	None in 6 preparations; 3–29 µM in 6 preparations	[121]
Instant coffee, 15 mg/mL, soon after preparation at 37 °C or 80 °C	Oxygen electrode	0.1 and 0.15 µM, respectively	[122]
Instant coffee (100 mg/mL, 24-h incubation)	Oxygen electrode	ca. 1000 ppm = 2.94 mM	[123]
Infusions of *Betula* leaves, *Polygonum*, *Lavandula* and *Tilia* inflorescence. 1-h incubation, room temperature	FOX assay (absorptiometric)	54, 37, 34 and 28 µM, respectively	[94]
Boiled homogenates of broad bean in deionized water, tap water and phosphate buffer, pH 7.4 (1 g/2 mL)	FOX assay (absorptiometric)	22.8, 28.1 and 76.0 µM, respectively	[91]
Boiled homogenates of broccoli, onion and leek (1 g/2 mL of phosphate buffer, pH 7.4)	FOX assay (absorptiometric)	25, 9 and 12 µM, respectively	[91]
Boiled black carrot homogenate, 1 g/5 mL in tap water and phosphate buffer, pH 7.4	FOX assay (absorptiometric)	8.7 and 55.0 µM, respectively	[92]
Commercial orange juices, Australia	FOX assay (absorptiometric)	2.26–8.88 µM (fresh), 4.99–14.51 µM (preserved)	[96]
Diluted Pinot and Merlot wines, 30-min incubation at 30 °C	Fluorimetric, with Ampliflu Red and HRP	12–16 µM	[99]
Xadrez (white) and Varna (red) wines, 3 d after opening	FOX assay (absorptiometric)	ca 18 and 32 µM, respectively	[100]
Ten beers, 4-fold diluted	FOX assay (absorptiometric)	0.4–7.9 µM	[102]
Brandy and whisky	FOX assay (absorptiometric)	2.2 and 21.6 µM, respectively	[102]
Dogwood, quince, hazelnut and apricot tinctures	FOX assay (absorptiometric)	8.7, 22.8, 23.3 and 48.3 µM, respectively	[102]

FOX, Ferrous Oxidation-Xylenol Orange; Hb, hemoglobin; HRP, horseradish peroxidase.

## Data Availability

No new data were created.

## Abbreviations

The following abbreviations are used in this manuscript:

DMEM	Dulbecco’s Modified Eagle’s Medium
DUOX	Dual oxidase
EGCG	Epigallocatechin gallate
ESR	Electron spin resonance
FOX	Ferrous Oxidation-Xylenol Orange
Hb	Hemoglobin
HRP	Horseradish peroxidase
Ma	Million years ago
NOX	NADPH oxidase
OPDV	N,N-diethyl-p-phenylenediamine
XO	Xanthine oxidase
